# Investigation into the causes of mortality in cetaceans and sirenian populations in the Andaman Sea, Thailand: A retrospective analysis spanning 2018–2023

**DOI:** 10.14202/vetworld.2024.2889-2898

**Published:** 2024-12-19

**Authors:** Chayanis Daochai, Peerapon Sornying, Narissara Keawchana, Sareepah Manmoo, Piyarat Khumraksa, Patchaporn Kaewmong, Santi Ninwat, Tipamat Upanoi, Pimwarang Sukkarun, Watcharapol Suyapoh

**Affiliations:** 1 Faculty of Veterinary Science, Prince of Songkla University, Songkhla, Thailand; 2 Dugong and Seagrass Research Station, Prince of Songkla University, Songkhla, Thailand; 3 Marine and Coastal Resources Research Center (Lower Andaman Sea), Trang, Thailand; 4 Marine and Coastal Resources Research Center (Upper Andaman Sea), Phuket, Thailand; 5 Department of Aquatic Animal Health Research and Clinic Center, Faculty of Veterinary Science, Rajamangala University of Technology Srivijaya, Nakhon Si Thammarat, Thailand

**Keywords:** dolphin, dugong, marine mammal, necropsy, stranding whale

## Abstract

**Background and Aim:**

Cetaceans and sirenians are endangered marine mammals that are threatened by stranding and mortality. In Thailand’s Andaman Sea, limited data exist on the causes and patterns of these events. This retrospective study investigated the characterization and underlying causes of cetacean and sirenian mortality events in the Andaman Sea from 2018 to 2023 using information from the Department of Marine and Coastal Resources, Thailand.

**Materials and Methods:**

Data on 363 stranded marine mammals, including both live and dead strandings, species types, carcass condition, and necropsy findings and diagnoses, were gathered and analyzed to classify and determine the main factors contributing to mortality, encompassing both direct human-related and non-direct human-related causes.

**Results:**

From 2018 to 2023, 231 cetaceans and 132 sirenians were documented, representing six families and 19 species. Of these animals, 18.18% (66/363) were stranded alive and 81.81% (297/363) were found dead. The most common species were dugong (*Dugong dugon*) and Indo-Pacific humpback dolphin (*Sousa chinensis*). Detailed postmortem analyses of 107 specimens showed that 17.76% (19/107) of deaths were anthropogenic, affecting 8 sirenians and 11 cetaceans. The majority of deaths were non-anthropogenic, involving 34 sirenians and 54 cetaceans. In addition, 223 stranded animals could not be fully assessed due to carcass condition.

**Conclusion:**

A high cetacean and sirenian mortality rate in the Andaman Sea can be attributed to non-anthropogenic factors. The dugong and Indo-Pacific humpback dolphin were the most frequently encountered species. This report enhances our understanding of marine mammal mortality in Thailand and underscores the need for improved health management and diagnostic responses.

## Introduction

Marine mammals, a diverse group comprising approximately 137 species, including cetaceans (whales, dolphins, and porpoises), pinnipeds (seals and sea lions), sirenians (manatee and dugong), and a few species of otters and bears, are facing significant threats worldwide [1]. Marine mammals play a crucial role in their ecosystems, contributing to the ocean’s food webs and the overall health of marine ecosystems through several roles, including nutrient cycling, predator-prey dynamics, and maintaining the balance of marine food webs [2, 3]. However, they encounter significant challenges from both anthropogenic and natural factors. Therefore, this underscores the urgent need for their conservation because their decline could lead to irreversible impacts on ecosystem functioning and services [4, 5].

Coastal regions in Thailand, including the Andaman Sea and Gulf of Thailand, are famous for their diverse marine mammals, such as whales, dolphins, and dugongs. Many of these animals are classified as protected under Thailand’s Wildlife Conservation and Protection Act, BE 2562, and are listed on the International Union for Conservation of Nature’s Red List of Endangered Species. Studies have identified 25 cetacean species and 1 sirenian species in Thai waters, with notable examples being the Indo-Pacific bottlenose dolphin and the pantropical spotted dolphin, both known for their high genetic diversity while also being protected in Thailand [6, 7]. The endangered dugong is found in significant numbers, particularly around Trang Province’s Muk and Talibong Islands in the Andaman Sea [8]. Genetic analysis has revealed higher genetic variation among dugongs in the Andaman Sea than among those in the Gulf of Thailand. This highlights the need for specific conservation initiatives because of potential concerns about breeding practices in the region [9]. The Andaman Sea is also home to various cetacean species, including common and rare marine mammals. Notable species include the Indo-Pacific bottlenose dolphin, Risso dolphin, and False killer whale. The unique genetic structures and presence of common and rare species in this region underscore the importance of marine conservation efforts [7, 10, 11].

Cetacean and sirenian stranding events are a global issue, with significant occurrences in Asia. From 2007 to 2019, Chinese waters experienced 1298 stranding events, with Taiwan reporting the most occurrences, while Indonesia has hotspots for single and mass strandings [12, 13]. Strandings are a complex issue influenced by various natural and anthropogenic factors. Globally, they are linked to climate and oceanographic variations, navigational errors due to magnetic anomalies, and anthropogenic activities [14, 15]. In Asia, particularly in Indonesian waters, strandings are often caused by strong ocean currents, extreme weather, decreased oxygen levels, and water salinity [16]. Parasitic infections also contribute to strandings [14]. Sirenians face threats from vessel strikes, entanglement in fishing gear, and habitat loss. Seasonal environmental parameters, such as wind-driven ocean circulation, influence prey distribution and cetacean movements [17]. In Thailand, out of 26 marine mammal species, at least 24 have been found stranded in the Gulf of Thailand and the Andaman Sea. A study by Pradip Na Thalang, Thongratsakul [18], documented 105 whales, 714 dolphins, and 103 dugongs that were found stranded between 2006 and 2015 in Thailand. Their research used spatial and temporal analyses to explore the stranding occurrences of endangered marine species based on stranding data and geographical records from 2006 to 2015. Previous studies by Adulyanukosol and Chantrapornsil [19] have highlighted the primary causes of stranding of endangered marine species, including topography, marine pollution, climate, natural toxicity, tidal disturbances, predator escape, chase victims, natural infection, misguided swarms, anthropogenic injuries, loss of echolocation, and navigation signaling problems.

Marine mammalian stranding records offer valuable opportunities for surveillance. This retrospective analysis aimed to achieve three main goals: to outline the epidemiology of marine mammal strandings along the Andaman Sea coastline, to examine marine mammal carcasses and classify pathological findings, and to identify the causes of strandings from both anthropogenic and non-anthropogenic perspectives. The findings of this investigation are important for developing conservation measures for cetaceans and sirenians in the Andaman Sea region. Understanding pathological data is crucial for future management and gaining insights into the overall health status and threats of these marine mammals.

## Materials and Methods

### Ethical approval

All data were obtained as part of routine diagnostic procedures. The program was not established as a research project; thus, no specific ethical approvals were required for this retrospective analysis.

### Study period and location

This study is based on data collected from January 2018 to December 2023. The analysis was conducted using data from two centers of the Marine and Coastal Resources Research Center: the Lower Andaman Sea (Trang) and the Upper Andaman Sea (Phuket). The analysis was completed in Febuary 2024.

### Experimental design and sample collection

Data were obtained from the Department of Marine and Coastal Resources of Thailand, specifically their database on marine mammals that are stranded or caught accidentally. In this retrospective analysis, reports on strandings and postmortem examinations were examined for all incidents involving marine mammals in the Ranong, Phang-nga, and Phuket areas covered by the Marine and Coastal Resources Research Center (Upper Andaman Sea), as well as in Krabi, Trang, and Satun under the purview of the Marine and Coastal Resources Research Center (Lower Andaman Sea) from 2018 to 2023.

At a minimum, information on the classification of marine mammals, along with the date, location, and type of incident, was documented for each stranding occurrence. Strandings refer to instances in which dead or live animals are found on the shore, found floating close to the coast, or caught unintentionally in fishing equipment. In most situations, efforts were made to rescue stranded animals. A Marine and Coastal Resources Research Center officer compiled reports of living and dead strandings. This study summarized the number of stranding events and individual animals categorized by family and species. Anatomic pathology is the primary diagnostic approach. A comprehensive postmortem examination of the stranded animals was conducted by a specialized marine veterinarian following a standardized procedure [20, 21]. Nutritional status was assessed based on a visual examination of anatomical parameters, such as the presence of certain prominent bones, the dorsoaxial muscular mass, and the absence or limited presence of fat, considering the species and the animal’s age. These findings led us to classify their nutritional status. The nutritional condition was assessed into cachexia and non-cachexia categories depending on the significant muscle loss and metabolic changes mentioned above [22].

### Carcass condition assessment and mortality categories

All carcasses were assigned a condition stage for decomposition at the time of necropsy: (1) (alive but subsequently died), (2) (carcass in good condition - fresh), (3) (fair condition with decomposition but intact organs, bloated), (4) (poor condition with advanced decomposition and bloating), or (5) (putrefaction - mummified carcass/skeletal remains). From animals in a very fresh or moderately autolyzed state, skin with blubber, skeletal muscle, lung, liver, kidney, and spleen were sampled for further study [21]. Categories of mortality identified include those linked to anthropogenic causes, non-anthropogenic causes, and those that were not determined. Anthropogenic causes include evidence of death during transport, pathological findings from foreign bodies, ship collisions, and interactions with fishing activities. On the other hand, non-anthropogenic causes are associated with pathologies linked to significant cachexia and non-cachexia conditions, perinatal disorders, and traumatic events, as categorized by Arbelo *et al*. [23].

## Results

### Epidemiology of marine mammalian strandings along the Andaman sea coast

During the sampling period, 363 marine mammals were found stranded, both alive and dead, along the coastline. This population comprised 231 cetaceans, among which 22.94% (53/231) were found alive and 77.06% (178/231) were found dead. Similarly, 132 sirenians were found, with 9.85% (13/132) found alive and 90.15% (119/132) found dead. These marine mammals were classified into six families: *Dugongidae*, *Delphinidae*, *Phocoenidae*, *Kogiidae*, *Balaenopteridae*, and *Physeteridae*, with one family remaining unidentified. The species within these families are listed in Table-1. The average annual stranding rate in this region during the specified period was calculated as 60.5 standings per year.

**Table-1:** A list of marine mammals stranded along the coastline of the Andaman Sea in Thailand from 2018 to 2023.

Common name	Family	Scientific name
Dugong	*Dugongidae*	*Dugong dugon*
Indo-Pacific humpback dolphin	*Delphinidae*	*Sousa chinensis*
Striped dolphin	*Delphinidae*	*Stenella coeruleoalba*
Spinner dolphin	*Delphinidae*	*Stenella longirostris*
Finless porpoise	*Phocoenidae*	*Neophocaena phocaenoides*
Indo-Pacific bottlenose dolphin	*Delphinidae*	*Tursiops aduncus*
Pantropical spotted dolphin	*Delphinidae*	*Stenella attenuata*
Irrwaddy dolphin	*Delphinidae*	*Orcaella brevirostris*
Fraser’s dolphin	*Delphinidae*	*Lagenodelphis hosei*
Dwarf sperm whale	*Kogiidae*	*Kogia sima*
Sperm whale	*Physeteridae*	*Physeter macrocephalus*
Bryde’s whale	*Balaenopteridae*	*Balaenoptera edeni*
Omura’s whale	*Balaenopteridae*	*Balaenoptera omurai*
Melon-head whale	*Delphinidae*	*Peponocephala electra*
Risso’s dolphin	*Delphinidae*	*Grampus griseus*
Pygmy sperm whale	*Kogiidae*	*Kogia breviceps*
False killer whale	*Delphinidae*	*Pseudorca crassidens*
*Stenella* spp.	*Delphinidae*	Cannot identify
Long-beaked common dolphin	*Delphinidae*	*Delphinus capensis*
Unknown	Unknown	Unknown

**Figure-1:**
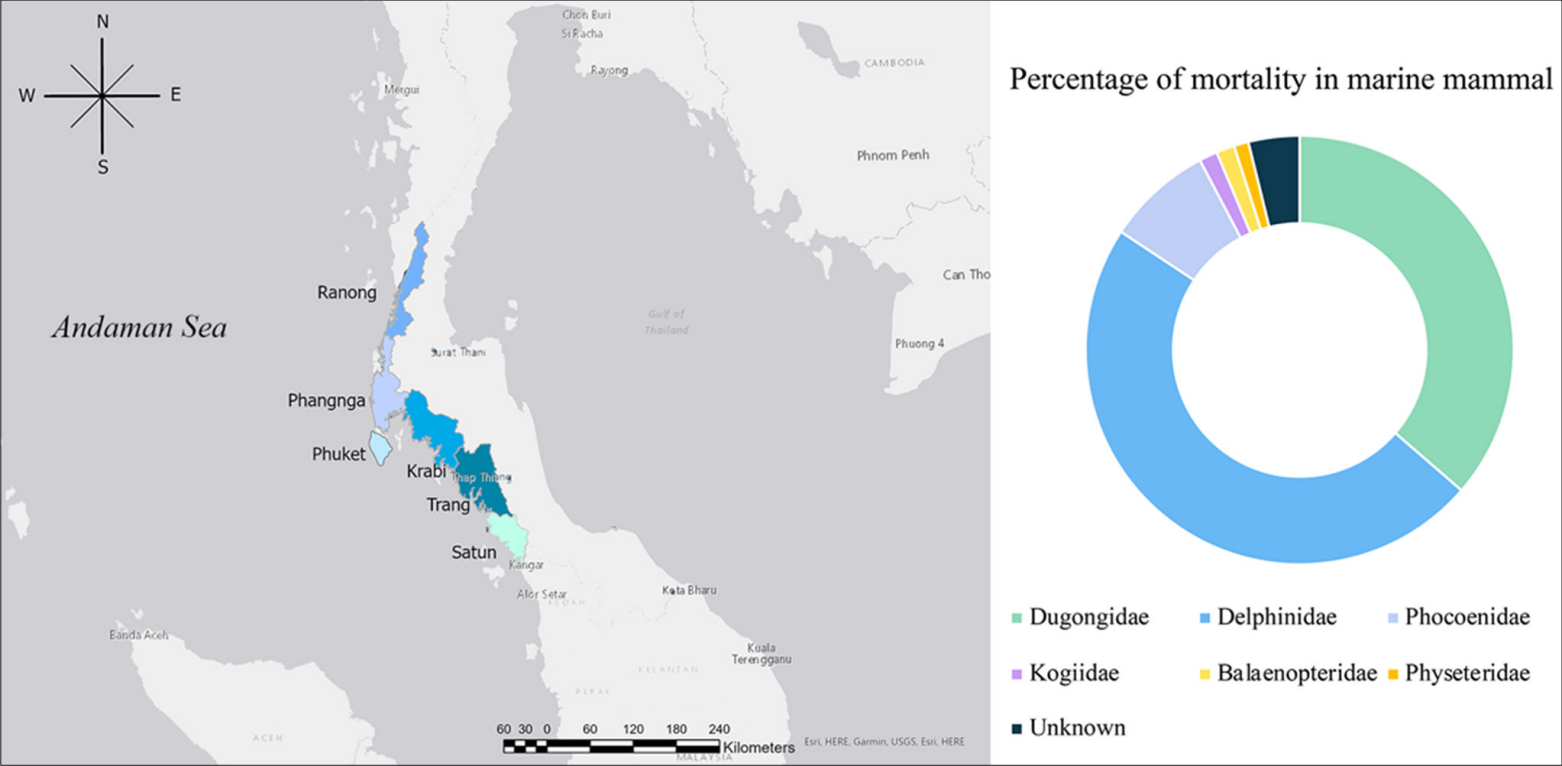
Percentage of mortality in cetacean and sirenian strandings along the Andaman Sea coastline of Thailand from 2018 to 2023. (Left) Map of Thailand showing 6 provinces along the coast of the Andaman Sea where marine mammal strandings were collected [Source: www.diva-gis.org]. (Right) most stranded species in this region were *Delphinidae* and *Dugongidae*.

Throughout the investigation period, the stranding mortality percentages of various marine mammalian families were analyzed. *Delphinidae* accounted for the highest proportion, comprising 47.93% (174/363) of the total recorded strandings. *Dugongidae* represented 36.36% (132/363), *Phocoenidae* 7.99% (29/363), both *Kogiidae* and *Balaenopteridae* constituted 1.38% (5/363) each, and *Physeteridae* each comprised 1.10% (4/363). Unidentified carcasses comprised 3.86% (14/363) of the total strandings (Figure-1).

At the species level, the top five most stranded marine mammals were *Dugong dugon*, accounting for 36.36% (132/363) of the total strandings, followed by *Sousa chinensis* at 12.95% (47/363), *Stenella coeruleoalba* at 11.57% (42/363), *Stenella longirostris* at 9.09% (33/363), and *Neophocaena phocaenoides* at 7.99% (29/363). The proportions of strandings among other marine mammalian species are shown in Table-2.

**Table-2:** Number and percentage of marine mammal stranding cases along the Andaman Sea coastline, Thailand (2018–2023).

Common name	Scientific name	Number of stranding cases	% of stranding cases
Dugong	*Dugong dugon*	132	36.36
Indo-Pacific humpback dolphin	*Sousa chinensis*	47	12.95
Striped dolphin	*Stenella coeruleoalba*	42	11.57
Spinner dolphin	*Stenella longirostris*	33	9.09
Finless porpoise	*Neophocaena phocaenoides*	29	7.99
Indo-Pacific bottlenose dolphin	*Tursiops aduncus*	18	4.96
Pantropical spotted dolphin	*Stenella attenuata*	12	3.31
Irrwaddy dolphin	*Orcaella brevirostris*	10	2.75
Fraser’s dolphin	*Lagenodelphis hosei*	6	1.65
Dwarf sperm whale	*Kogia sima*	4	1.10
Sperm whale	*Physeter macrocephalus*	4	1.10
Bryde’s whale	*Balaenoptera edeni*	3	0.83
Omura’s whale	*Balaenoptera omurai*	2	0.55
Melon-head whale	*Peponocephala electra*	2	0.55
Risso’s dolphin	*Grampus griseus*	1	0.28
Pygmy sperm whale	*Kogia breviceps*	1	0.28
False killer whale	*Pseudorca crassidens*	1	0.28
*Stenella* spp.	Cannot identify	1	0.28
Long-beaked common dolphin	*Delphinus capensis*	1	0.28
Unknown	Unknown	14	3.86

### Investigation of stranded marine mammals

We investigated the quantity and condition of carcasses from live and dead stranded marine mammals along the Andaman Sea coastline of Thailand by examining necropsy reports. The total number of stranding cases was 363, comprising 231 cetaceans and 132 sirenians. Among these, 66 were live strandings. Half of the live-stranded individuals died during treatment, whereas the other half were successfully released back into the Andaman Sea. Thirty-three carcasses of marine mammals that died after rescue and rehabilitation were included in the total number of carcasses investigated, amounting to 330 carcasses, which included 205 cetaceans and 125 sirenians (Table-3).

**Table-3:** Summary of live and dead stranded cetaceans and sirenians, mortality post-treatment, and total carcasses.

The type of carcass	Total cases	Number of stranded marine mammals			Total number of carcasses

		Live stranding	Dead stranding	Die after live stranding	
Cetacean	231	53	178	26	205
Sirenian	132	13	119	7	125
Total	363	66	297	33	330

To investigate the quality of the carcasses, following the marine mammal necropsy guide procedure, carcasses were categorized into five groups: (1) alive but subsequently died; (2) carcass in good condition (fresh); (3) carcass fair (decomposition but organ intact, bloated); (4) carcass poor (advanced decomposition, bloated); (5) putrefaction (mummified carcass, skeletal remains). Our study found that the majority of carcasses were in advanced decomposition, comprising 52.12% (172/330), with 110/204 cetaceans and 62/126 sirenians. Only 30.30% (100/330) of the stranded marine mammals were in the live stage to the fresh condition (1 and 2). These carcasses included 59/204 cetaceans and 41/126 sirenians. In addition, one sirenian carcass was reported to have been lost at sea while technical staff arrived to collect it. The carcass stages are described in Table-4.

**Table-4:** Distribution and percentage of carcasses at different stages of premortem and postmortem findings in stranding cases along the Andaman Sea coastline, Thailand (2018–2023).

The types of carcasses (total number)	Number of carcasses at various stages among the animal groups (% of carcass)					

	1 (%)	2 (%)	3 (%)	4 (%)	5 (%)	Loss* (%)
Cetacean (204)	26 (12.74)	33 (16.17)	26 (12.74)	110 (53.92)	9 (4.41)	0 (0.00)
Sirenian (126)	7 (5.55)	34 (26.96)	22 (17.46)	62 (53.17)	0 (0.00)	1 (0.79)
Total (330)	33 (10.00)	67 (20.30)	48 (14.54)	172 (52.12)	9 (2.72)	1 (0.30)

1 alive but subsequently died; 2, carcass in good condition (fresh); 3, carcass fair (decomposition but organ intact, bloated); 4, carcass poor (advanced decomposition, bloated); 5, putrefaction (mummified carcass, skeletal remains).

* Loss refers to the disappearance of a marine mammal carcass in the sea when the technical staff arrived to collect it

### Necropsy reports and pathological categories

Excluding cases of cetaceans and sirenians released back into the sea, 330 cetacean and sirenian carcasses were included in this study. The causes of death for 223 marine mammal carcasses could not be determined due to poor carcass condition, technical errors, or accidental loss of the carcasses. Consequently, only 107 marine mammal carcasses underwent thorough necropsy procedures, comprising 39.25% (42/107) sirenians and 60.74% (65/107) cetaceans. All relevant pathological categories were retrospectively extracted from the necropsy documents. The causes of death were divided into major categories: Anthropogenic origin and non-anthropogenic (natural) origin. Among the 107 marine mammal carcasses, 17.76% (19/107) were attributed to anthropogenic origins, with 8 being sirenians and 11 cetaceans. Meanwhile, 82.24% (88/107) of the carcasses were of natural origin, comprising 34 sirenians and 54 cetaceans.

Our study categorized pathological findings from necropsy reports into those associated with human activities and those not associated with human activities. We identified deaths during transport, foreign body pathology, ship collisions, and interactions with fishing activities among the human-related categories. In contrast, the non-human-related categories included pathology associated with significant cachexia, pathology associated with non-cachexia, perinatal pathology, and intra-/interspecific traumatic interactions. In the anthropogenic classification, one marine mammal [0.93% (1/107)] was recorded as died during transportation, with this cause observed only once in *S. coeruleoalba*. Similarly, one marine mammal [0.93% (1/107)], *S. chinensis*, was affected by foreign body pathology. Ship collisions accounted for 11.21% (12/107) cases involving the following species: *D. dugon* (6/12), *S. coeruleoalba* (3/12), *S. chinensis* (1/12), *S. longirostris* (1/12), and *Kogia sima* (1/12). Interactions with fishing activities were responsible for 4.67% (5/107) cases, including the following species: *D. dugon* (2/5), *S. chinensis* (1/5), *S. coeruleoalba* (1/5), and *Balaenoptera omurai* (1/5). Regarding the non-anthropogenic classification, the most prevalent category was pathology associated with non-cachexia, comprising 63.55% (68/107) cases. Pathology linked to significant cachexia was the second most common cause, accounting for 14.02% (15/107) cases, which included the following species: *D. dugon* (6/15), *S. chinensis* (5/15), *N. phocaenoides* (2/15), *Tursiops aduncus* (1/15), and *Stenella attenuata* (1/15). Intra-/interspecific traumatic interactions accounted for 3.74% (4/107) cases, all of which were found in *D. dugon*. Perinatal pathology was responsible for 0.93% (1/107) case, which also occurred in *D. dugon*. A detailed analysis of the predominant causes of marine mammalian strandings for each species is presented in Table-5.

**Table-5:** Anthropogenic and non-anthropogenic classifications of stranded marine mammals from the Andaman Sea. Cetacean and sirenian strandings along the Andaman Sea coastline of Thailand from 2018 to 2023 were analyzed, focusing on the number of cases per species across various pathological categories.

Species	Anthropogenic				Non-anthropogenic (natural)				ND	Total
	
	DT	FBP	SC	IFA	PASC	PANC	PP	IITI		
*Dugong dugon*	0	0	6	2	6	23	1	4	83	125
*Sousa chinensis*	0	1	1	1	5	7	0	0	30	45
*Stenella coeruleoalba*	1	0	3	1	0	9	0	0	16	30
*Stenella longirostris*	0	0	1	0	0	11	0	0	16	28
*Neophocaena phocaenoides*	0	0	0	0	2	3	0	0	24	29
*Tursiops aduncus*	0	0	0	0	1	2	0	0	12	15
*Stenella attenuata*	0	0	0	0	1	3	0	0	6	10
*Orcaella brevirostris*	0	0	0	0	0	3	0	0	7	10
*Lagenodelphis hosei*	0	0	0	0	0	3	0	0	3	6
*Kogia sima*	0	0	1	0	0	2	0	0	1	4
*Physeter macrocephalus*	0	0	0	0	0	0	0	0	4	4
*Balaenoptera edeni*	0	0	0	0	0	0	0	0	3	3
*Balaenoptera omurai*	0	0	0	1	0	0	0	0	1	2
*Peponocephala electra*	0	0	0	0	0	1	0	0	0	1
*Grampus griseus*	0	0	0	0	0	1	0	0	0	1
*Kogia breviceps*	0	0	0	0	0	0	0	0	1	1
*Pseudorca crassidens*	0	0	0	0	0	0	0	0	0	0
*Stenella* spp.	0	0	0	0	0	0	0	0	1	1
*Delphinus capensis*	0	0	0	0	0	0	0	0	1	1
Unknown	0	0	0	0	0	0	0	0	14	14
Total	1	1	12	5	15	68	1	4	223	330
% of all cases	0.30	0.30	3.64	1.52	4.55	20.61	0.30	1.21	67.58	
% without ND	0.93	0.93	11.21	4.67	14.02	63.55	0.93	3.74		

These categories include. DT=Dead during transport, FBP=Foreign body pathology, SC=Ship collisions, IFA=Interaction with fishing activities, PASC=Pathology associated with significant cachexia, PANC=Pathology associated with non-cachexia, PP=Perinatal pathology, IITI=Intra-/interspecific traumatic interactions, and ND=Not determined

### Etiological diagnosis of anthropogenic and nonanthropogenic origins

The predominant pathological conditions affecting cetaceans in the Andaman Sea were identified by examining 65 carcasses. Septicemia of unknown origin was the most prevalent condition observed in 19 carcasses. The common signs of septicemia include carcass redness, vascular engorgement, generalized lymphadenopathy, and pulmonary congestion with or without pneumonia. In addition, multifocal granulomatous or abscess formations and areas of pulmonary necrosis were noted. Pulmonary inflammation without cachexia was observed in 15 carcasses, characterized by increased lung weight, patchy mottled red/brown regions (consolidation), petechial hemorrhage, suppuration, fibrin formation, granuloma, and aspiration. Calf separation from the mother with cachexia features was identified in 6 carcasses, with milk-like material found in the gastric contents of some calves and empty stomachs in others. Ship collisions were also implicated in 6 carcasses, displaying severe multiple bone fractures, bruises, and traumatic wounds on the skin.

In contrast, sirenian cases examined from 42 carcasses were predominantly characterized by severe parasitic infections without cachexia, occurring in 6 cases. These cases primarily involved severe nematode compaction in the gastrointestinal tract, with some cases exhibiting granuloma formation in the gastric wall. Collisions with vessels were also reported in 6 carcasses, while septicemia without cachexia was documented in 4 carcasses, displaying gross lesions similar to cetaceans but commonly associated with gastroenteropathy. Blunt trauma caused by stingray spines and dugong tusks was another significant cause, observed in 4 carcasses. Pneumonia without cachexia and mother-calf separation was each observed in 3 carcasses. The detailed etiological pathologies of both cetaceans and dugongs are presented in Table-6.

**Table-6:** Cetacean and sirenian strandings along the Andaman Sea coastline of Thailand from 2018 to 2023: Quantification of etiological diagnoses across various pathological categories.

Pathological category	Etiologic diagnosis	No. of cetacean	No. of sirenian	Total number of cases
Anthropogenic origin				
Dead during transport	Suspect capture myopathy	1	0	1
Foreign body pathology	Foreign body ingestion	1	0	1
Collisions with vessels	Blunt trauma, bruise, fracture	6	6	12
Interactions with fishing activities	Bycatch	0	1	1
	The drowning resulted from the entrapment of a fishing tool	3	1	4
Natural origin				
Pathology associated with cachexia	Parasitic infestation and disease	1	1	2
	Senile changes	2	0	2
	Pneumonia, pneumonitis	1	2	3
	Mother–calf separation	6	3	9
Pathology associated with non-cachexia	Parasitic infestation and disease	5	6	11
	Pneumonia, pneumonitis	15	3	18
	Congestive heart failure	1	1	2
	Myocardial infarction	0	1	1
	Chronic kidney disease	0	1	1
	Diaphragmatic hernias	0	1	1
	Peritonitis	0	2	2
	Meningoencephalitis of unknown origin	1	0	1
	Unknown origin of septicemia	19	4	23
	Unknown origin enteritis	1	1	2
	Intestinal volvulus and necrosis	0	1	1
	Pyometra	1	0	1
	Neoplasia	1	2	3
Traumatic intra- and interspecific interactions	Blunt trauma caused by a stingray spine, bite scars, and/or tooth-rake marks	0	4	4
Perinatal pathology	Still birth	0	1	1
	Total	65	42	107

## Discussion

In Thailand, marine mammals are primarily classified as endangered because of reports of declining populations [24]. Although spatial and temporal analyses have been conducted to investigate the stranding of endangered marine species (whales, dolphins, dugongs, and sea turtles) in Thailand, research on pathology is currently lacking [18]. This study is the first report on stranded marine mammal data in the Andaman Sea, focusing on the investigation of carcasses and pathological categories. The study recorded 923 stranded marine mammals, including whales, dolphins, and dugongs, in the Andaman Sea and Gulf of Thailand from 2006 to 2015. A previous study reported that 43.06% of these animals were stranded along the Thai coast of the Andaman Sea. However, a direct comparison with our study’s timeframe is not available [18]. Our findings revealed 363 stranded animals documented in the Andaman Sea region from 2018 to 2023. At the species level, Thailand’s most frequently stranded marine mammals include finless porpoises, Irrawaddy dolphins, Indo-Pacific humpbacked dolphins, dugongs, and bottlenose dolphins, with a notable number of Bryde’s whales also observed [18]. Our study, focusing on the Andaman Sea area, found that *D. dugon* was the most frequently stranded species, followed by *S. chinensis*, *S. coeruleoalba*, *S. longirostris*, and *N. phocaenoides*. The number of dugong strandings in the Andaman Sea was higher than the overall stranding data for Thailand [25], the Philippines [26] and the surrounding areas of the Malaysia, Indonesia, and Thailand Sea [27]. In comparison with Southeast Asian countries, the Philippines recorded the highest frequency of marine mammal strandings from 1999 to 2009. The results showed that most of these strandings involved cetaceans, with the most common species being *S. longirostris*, *Globicephala macrorhynchus*, and *Peponocephala electra*. These stranding events frequently involved unique species, such as Longman’s beaked whale, short-finned pilot whale, and melon-headed whale [25]. Similarly, Indonesia reported that the majority of strandings involved cetaceans, including *S. longirostris*, *Tursiops* ssp., *Physeter macrocephalus*, *G. macrorhynchus*, and *Megaptera novaeangliae* [28, 29]. In Malaysia, the most commonly stranded marine mammal species vary depending on the year of recovery. In 2001 and 2004, dolphins were the most frequently stranded species, with *Orcaella brevirostris*, *S. chinensis*, *T. aduncus*, *S. longirostris*, and *S. attenuata* being the most commonly recorded. In contrast, in 1996 and 2001, the *D. dugon* was the most commonly stranded marine mammal [30]. This highlights the distinct presence of these species in specific regions and underscores the importance of marine conservation efforts.

Marine mammal stranding events provide valuable insights into the health and mortality of cetacean populations. Carcass condition is crucial in determining the cause of death, with fresh carcasses allowing for comprehensive necropsies to determine infections or trauma as causes of death, while severely decomposed carcasses limit diagnostic capabilities [31]. A study along the Ligurian coast found that 19.27% (16/83) of carcasses were severely decomposed, while research in the Canary Islands reported a rate of 28.26% (39/138) for advanced or very advanced decomposition. [23]. In contrast, our study observed a notably higher proportion of carcasses, accounting for 52.12% (172/330), in an advanced stage of decomposition. We attribute this elevated decomposition rate to various environmental factors, including seasonal variations, temperature gradients, shallow water habitats, and warm water conditions [32]. However, previous study by Giorda *et al*. [33] has suggested that necropsy and histopathological investigations on decomposing carcasses should be conducted during stages 2–3, as this is when determining the cause of death becomes particularly challenging.

Marine mammalian populations in Thailand are declining because of natural and human-induced factors [34]. In our study, the postmortem results revealed that 17.76% of the mortalities had anthropogenic origins, with 8 from Sirenians and 11 from Cetaceans. Anthropogenic activities, including bycatch and fishing gear entanglement, significantly contribute to cetacean mortality in various locations. For example, 12% of marine mammal strandings that occur in the St. Lawrence Estuary show evidence of anthropogenic trauma. In the Canary Islands, human activities account for 19% of cetacean deaths. Bycatch is the leading cause of death for harbor porpoises in the North Sea; fisheries’ bycatch is primarily responsible for a strikingly high percentage (65.2%) of small cetacean deaths in southern Brazil [33, 35–37]. Natural causes, such as infectious diseases, predation, and neonatal pathology, are major contributors to cetacean death in various regions. In the Canary Islands, around 81% (169/208) of cetacean deaths are linked to natural pathologies, whereas in the Netherlands, infectious diseases are the primary cause of fatalities in harbor porpoise [38, 39]. In our study, the majority [82.24% (88/107)] of incidents were non-anthropogenic, which included 34 sirenians and 54 cetaceans.

Our examination of 65 cetacean carcasses retrieved from the Andaman Sea revealed that septicemia, non-cachexic pulmonary inflammation, and the separation of calves from their mothers were frequently observed as abnormal conditions. Cetaceans often suffer from infectious and inflammatory illnesses when stranded, and septicemia and pulmonary inflammation are commonly observed. Instances of septicemia have been documented in multiple cases, such as a Southern right whale calf with *Streptococcus dysgalactiae*, a Cuvier’s beaked whale with *Morganella morganii*, and a bottlenose dolphin with *Erysipelothrix rhusiopathiae* [40, 41]. These opportunistic pathogens frequently originate from compromised health due to underlying factors, such as parasitic infestations and environmental stressors. Pneumonia is another prevalent cause of death in stranded cetaceans, often occurring alongside nematode infections and parasitism. This leads to severe lung damage and secondary bacterial infections, as observed in species such as harbor porpoises. Along the Parana coast of Brazil, bronchointerstitial pneumonia associated with parasitism has been documented in different types of cetaceans [42, 43]. Our sirenian cases were mainly defined as parasitic infections without the presence of cachexia, followed by septicemia and pneumonia. Several studies have documented these, highlighting their occurrence and potential impact on these marine mammals [44–46]. Dugongs in the Philippines [44] and manatees in USA [47] and Puerto Rico [48] were infected with various parasites during necropsies. This included roundworms, trematodes, and helminths such as *Heterocheilus tunicatus*, *Chiorchis fabaceous*, *Pulmonicola cochleotrema*, *Chiorchis groschi*, and *Pulmonicola cochleotrema*. *Toxoplasma gondii* has also been identified as a significant emerging pathogen causing severe inflammatory lesions in the gastrointestinal tract and heart of Antillean manatees in Puerto Rico [49]. Our investigation into infectious agents is limited by a lack of data, which may be attributed to constraints such as the financial resources of the research center, logistical challenges, and the quality of tissue samples. The observed calf separation in our study is a significant issue. The physiological and behavioral development of calves and the health of their mothers play a role in the separation of stranded marine mammals. It has been reported that underdeveloped muscles due to incomplete postnatal muscle biochemistry maturation can lead to separation during stressful events, such as fishery operations [50, 51].

The leading global anthropogenic causes of cetacean mortality include bycatch, ship collisions, marine debris ingestion, gunshots, and entanglement in fishing gear. In our study, the primary cause was ship collision, followed by a small amount of fishing-related activities. Studies have found that vessel collisions cause severe harm or fatalities in species such as the North Atlantic right whale [52] and fin whales [53]. In the North Atlantic region, ship accidents caused 35.5% of right whale fatalities between 1970 and 1999, often leading to immediate or delayed death due to extensive trauma. Ship collisions were also a primary factor in the mortality of *Physeteridae* in the Canary Islands, accounting for 66.6% of deaths within this family [52, 53]. Our research indicates that ship collisions significantly impact dugong, striped dolphin, and spinner dolphin. These species often share habitats with areas of high maritime traffic, such as coastal regions and estuaries where vessel activity is concentrated [54]. Monitoring and mitigation measures are important for reducing the risk of vessel collisions with cetacean communities in the Andaman Sea. This is a growing concern [55]. Although only 17.76% (19/107) of cases were linked to direct human-induced factors in this research, the adverse effects of anthropogenic stressors may have been underestimated. While not causing immediate animal fatalities, human activities can cause long-term sublethal consequences for cetaceans, including chronic stress, disruption in predation patterns, and physiological stress-related pathology in Taiwanese water [56].

## Conclusion

This study provides valuable insights into mortality patterns, particularly disease-related deaths among cetaceans and sirenians. The increased incidence of natural diseases may be attributed to tropical environments and climate changes. Nevertheless, attention must be directed toward human-induced mortality, particularly in dugongs, which are susceptible to collisions with vessels and interactions with fishing activities. However, further research focusing on detailed disease investigations is imperative to tackle the challenges associated with financing, logistics, and the availability of diagnostic tools. This will facilitate a deeper understanding of the critical threats to marine mammals’ well-being, optimal ocean health, and implications for human health.

## Authors’ Contributions

CD: Conceptualization, data curation, investigation, methodology, project administration, supervision, validation, visualization, and writing and editing. PSo, NK, SM, SN, and TU: Conceptualization. PKh and PKa: Methodology. PSu: Data curation, validation, writing, review, and editing. WS: Conceptualization, data curation, investigation, methodology, project administration, supervision, validation, visualization, and writing and review. All authors have read and approved the final manuscript.
